# Implantation cochléaire au Sénégal: enjeux, limites et perspectives

**DOI:** 10.11604/pamj.2023.45.12.39881

**Published:** 2023-05-04

**Authors:** Cheikh Ahmédou Lame, Abdou Sy, Birame Loum

**Affiliations:** 1 Service d'Oto-Rhino-Laryngologie (ORL), Hôpital Principal de Dakar, Dakar, Sénégal,; 2 Service d'Oto-Rhino-Laryngologie (ORL), Hôpital Pour Enfants de Diamniadio, Rufisque, Sénégal

**Keywords:** Surdité, implantation cochléaire, Afrique, Sénégal, Hearing loss, cochlear implantation, Africa, Senegal

## Abstract

L´implant cochléaire est une prothèse auditive électronique qui s´adresse aux surdités sévères à profondes bilatérales. Il permet de stimuler directement les fibres du nerf cochléaire en court-circuitant les cellules ciliées. Cette technologie très performante apparue depuis 60 ans, s´est largement répandue à travers le monde et est régulièrement utilisée dans la réhabilitation auditive. Dans les pays en voie de développement, l´adoption et le développement de cet outil accusent encore du retard. Les auteurs analysent dans cet article les facteurs qui retardent la pénétration de l´implant cochléaire au Sénégal.

## Abstract (en)

A cochlear implant is an electronic prosthesis used in the rehabilitation of profound bilateral hearing loss. It directly stimulates cochlear nerve fibers by bypassing the hair cells. This high-performance technology, which appeared 60 years ago, has spread widely throughout the world and is regularly used in hearing rehabilitation. In developing countries, the adoption and development of this tool are still lagging behind. The authors analyze factors delaying the penetration of cochlear implants in Senegal.

## Perspectives

L´implant cochléaire (IC) est une prothèse auditive implantée par voie chirurgicale, qui stimule électriquement les origines du nerf auditif en court-circuitant l´organe sensoriel de Corti. Il s´adresse à la quasi-totalité des surdités sévères à profondes bilatérales aussi bien de l´enfant que de l´adulte [1-3]. Cette technologie innovante est reconnue comme performante et en constante évolution [4,5]. Si dans les pays industrialisés, les implantations cochléaires se sont développées depuis le début des années 90; en Afrique Subsaharienne, l´utilisation de cette technologie est encore à l´état de balbutiements. L´objectif de ce travail est de faire l´état des lieux au Sénégal, d´identifier les obstacles et d´envisager l´avenir de l´implantation cochléaire dans notre pays.

**Historique de l´implantation cochléaire:** le premier implant cochléaire monoélectrode a été développé en 1957 en France par Charles Eyriés, otologiste, et André Djourno, physicien [1,4]. C´est en 1961, que W. House aux États Unis, améliora la première version de Eyriés. Il introduit pour la première fois l´électrode dans la fenêtre ronde. De nombreux patients furent ainsi implantés. La version multiélectrode de l´implant cochléaire vit sa naissance en 1977 à Paris, où il fut développé par l´équipe de l´Hôpital Saint Antoine, avec 3 électrodes. Puis ce prototype a été amélioré en Autriche, où il passa à 12 électrodes, avec une réduction de volume de l´émetteur extérieur.

Les australiens contribuèrent considérablement aussi au développement technologique de l´IC en travaillant sur la miniaturisation et la digitalisation de l´implant. Ensuite, les américains participèrent dans les années 90 à l´amélioration du signal sonore [1]. L´approbation par la FDA (Food and Drug Administation) du premier IC multiélectrode fut adoptée en 1985 aux Etats Unis [4,5]. Depuis lors, la technologie s´est améliorée et les indications s´élargissent. Les progrès, aussi, continuent. Aujourd´hui l´IC est considéré comme une technologie fiable et efficace dans la réhabilitation auditive des surdités sévères à profondes bilatérales [4,5]. Au Sénégal, la première chirurgie d´implantation cochléaire a eu lieu en 2011. Elle a été réalisée au niveau du service universitaire avec la collaboration d´otologistes espagnols. Depuis lors, 24 patients ont été implantés dans le pays au niveau de 3 centres différents (Hôpital Fann, Hôpital Principal de Dakar, Hôpital pour Enfants de Diamniadio). Dans notre pays, le développement de cette chirurgie se heurte à plusieurs difficultés.

**Limites épidémiologiques:** selon une étude récemment publiée dans la revue *The Lancet*, par le Global Burden Disease, le nombre de personnes souffrant dans le monde de déficience auditive plus ou moins prononcée est de 1,5 milliards. Parmi elles, 430 millions ont besoin de réhabilitation auditive [6]. La même étude prévoit un taux de croissance de 56,1% dans les 30 prochaines années, soit 2,5 milliards de personnes qui seront atteintes de déficience auditive plus ou moins prononcée, et environ 700 millions qui auront besoin de réhabilitation auditive [6]. L´Organisation Mondiale de la Santé (OMS) estime qu´entre 2010 et 2020 la prévalence de la surdité incapacitante était de 1,9% pour les enfants de moins de 14 ans et de 38% pour les personnes âgées de 65 ans et plus [6]. Cependant, force est de constater que dans les pays en développement, les données et études sur la surdité sont en nombre très limité [4,7]. Au Sénégal, il n´existe toujours pas de données épidémiologiques nationales précises sur la prévalence et l´incidence de la surdité. Ce déficit statistique freine considérablement la sensibilisation sur la surdité, dont l´ampleur reste sous-estimée. Cela explique aussi l´orientation des stratégies nationales de lutte contre les maladies vers d´autres pathologies tels le paludisme, le VIH, la tuberculose, le cancer.

**Insuffisance du dépistage:** la forte prévalence de la surdité, l´existence de tests de dépistage fiables, la possibilité d´une prise en charge précoce et, surtout, le bénéfice de cette prise en charge précoce, justifient la mise en place de stratégies de dépistage de la surdité. Dans la plupart des pays développés, le dépistage de la surdité est systématisé à la naissance [8]. C´est le cas en France, où le dépistage de la surdité est rendu obligatoire depuis 2012, par un arrêté ministériel [9]. Deux tests ont été validés et sont utilisés pour le dépistage. Il s´agit des otoémissions acoustiques provoquées (OEAP) et des potentiels évoqués auditifs automatisés (PEAA). Ces deux tests, assez accessibles sur le plan financier, sont simples d´utilisation et peuvent être réalisés par du personnel non spécialisé [9].

Ces tests bien qu´existants dans notre pays, sont très peu vulgarisés en Afrique Subsaharienne [8]. Le coût du dépistage au Sénégal est évalué entre 30 et 45000 FCFA (45 et 70 Euros). Leur utilisation se heurte encore au déficit en sensibilisation, à la barrière des croyances socio-culturelles mais aussi et surtout, à l´absence de mesures efficaces de suivi et de prise en charge des patients dépistés. Des initiatives locales commencent à être développées au niveau de certaines structures sanitaires. Cependant, des décisions au niveau central sont nécessaires pour la vulgarisation voire la systématisation du dépistage de la surdité à la naissance.

**Limites dans le diagnostic:** l´étape diagnostique correspond à la réalisation d´un bilan complet de l´audition après dépistage. Ce bilan permet de poser le diagnostic de surdité en précisant le type d´atteinte (surdité de transmission, surdité de perception, ou surdité mixte) ainsi que le degré (surdité légère à profonde) [9,10]. Le bilan oriente aussi vers le site lésionnel (cochlée ou voies auditives), en fonction de la présence ou non des OEAP et de l´aspect des courbes d´enregistrement des PEA [11]. Il passe d´abord par un interrogatoire complet, suivi d´un examen otologique minutieux, insistant sur l´aspect des tympans [10,12]. Les explorations fonctionnelles (PEA, OEAP, Auditory Steady State Responses (ASSR), impédancemétrie) complètent l´exploration [10,12]. La biologie (sérologies, génétique) et l´imagerie contribuent à préciser le diagnostic étiologique [10,12,13]. Tous ces outils diagnostiques sont disponibles au Sénégal, même s´ils restent concentrés dans la capitale dakaroise avec un coût financier significatif surtout chez les populations ne disposant pas de couverture médicale. Le bilan diagnostique complet de la surdité a été estimé à environ 500.000F CFA (765 euros). Cela correspond à 5,5 fois le salaire mensuel moyen brut au Sénégal.

**Limites dans la chirurgie:** la chirurgie otologique reste très peu développée dans notre pays avec en moyenne 60 médecins ORL dont 10 chirurgiens otologistes pour une population de 18.000.000 d´habitants. Parmi ceux-ci, un petit nombre a l´expérience de la chirurgie de l´implantation cochléaire. Le même constat est fait au Nigeria, où pour une population de 160 millions d´habitants, Adedeji *et al*. trouvent 200 chirurgiens ORL et 30 orthophonistes [8]. Ces chiffres, très différents dans les pays développés, sont respectivement de 3000 et 24000 en France pour 68 millions d´habitants, 12000 et 117000 aux USA pour 332 000 000 d´habitants [14]. Au Sénégal, le plateau technique pour l´implantation cochléaire est disponible. Mais, pour le moment, seuls 3 centres ont déjà réalisé ce type d´intervention.

**Obstacles liés à la rééducation orthophonique:** la rééducation orthophonique constitue la pierre angulaire dans le processus d´acquisition du langage oral après implantation cochléaire [15,16]. Ainsi, un plan de rééducation élaboré est nécessaire pour répondre aux besoins de l'enfant et faciliter les résultats souhaités. Ce plan implique une approche multidisciplinaire avec la collaboration de l´otologiste, de l´audiologiste et de l´orthophoniste [17]. Très peu d´orthophonistes existent dans le pays. A ce jour, ils sont moins de dix; et ils doivent s´occuper de la rééducation de tous les patients pour toutes les spécialités.

**Fardeau socio-économique:** il est démontré que le statut socioéconomique influence l´accès à l´implantation cochléaire et détermine la qualité des résultats obtenus, l´acquisition de la parole et du langage [18-24]. Le coût de la prise en charge de cette chirurgie (bilan pré-implant, prix de l´implant, suivi orthophonique) demeure très onéreux [21,22,25,26]. Dans les pays développés et dans certains pays en voie de développement, ce coût est pris en charge par les systèmes de sécurité sociale ou les compagnies d´assurance [9,23]. Au Sénégal, ce coût est actuellement estimé entre 15 et 20 millions de francs CFA (23000 et 30000 euros). Pour rappel, en 2019, le salaire mensuel moyen brut était estimé à 89 730 francs CFA (138 euros), selon le Ministère de l'Économie, du Plan et de la Coopération du Sénégal. Les examens complémentaires et la chirurgie peuvent généralement être pris en charge par l´employeur ou les compagnies d´assurances pour les salariés et leurs familles. Toutefois, l´acquisition de l´implant qui représente la partie la plus coûteuse, reste à la charge du patient. Un soutien financier aux candidats à l´IC et à leurs familles, est nécessaire, mais n'est pas toujours réalisable dans notre contexte de pratique [27].

**Perspectives:** les centres ORL se multiplient dans tout le pays et le plateau technique s´améliore. Les praticiens ORL s´intéressent davantage à la chirurgie otologique, qui reste encore peu développée en Afrique subsaharienne. Les cours de dissection de l´os temporal qui constituent un moyen essentiel d´apprentissage en otologie [28,29] sont désormais organisés à Abidjan (en Côte d´Ivoire) et à Thiès (Sénégal). De même, une école d´orthophonie a vu le jour au Sénégal il y´a 2 ans. La première promotion d´orthophonistes, formés sur place, est attendue l´année prochaine. Des associations de lutte contre la surdité et de promotion de l´implantation cochléaire ont été mises sur pied. Leur mission est, entre autres, de mener un plaidoyer en direction des autorités étatiques pour la prise en charge de la surdité.

## Conclusion

Cette analyse situationnelle nous permet d´identifier les obstacles persistants au développement de l´implantation cochléaire au Sénégal. Elle souligne les grands écarts à combler dans la sensibilisation des populations, des décideurs mais également dans la formation des chirurgiens et l´équipement des structures sanitaires. La vulgarisation du dépistage néonatal de la surdité permettra d´apprécier l´incidence de ce handicap, d´avoir des données statistiques fiables et de convaincre les autorités sanitaires quant à la nécessaire prise en charge de la réhabilitation des surdités sévères à profondes.
